# Books and Authors

**Published:** 1910-07

**Authors:** 


### BOOKS AND AUTHORS.

---

**Diseases of the Genitourinary Organs.** By **Edward L. Keyes,** Jr., M.D., Ph.D., Clinical Professor of Genitourinary Surgery in the New York Polyclinic Medical School. Octavo, pp. 975. With 195 illustrations in the text and 7 plates, four of which are colored. New York and London. D. Appleton & Co. 1910. (Cloth, $5.00.)

In the year 1874 the treatise of Van Buren and Keyes appeared, taking at once a leading place in the literature of diseases of the genitourinary organs. The present edition, prepared under the authorship of the younger Keyes, may be regarded as a continuation of the Van Buren and Keyes work—that is, as a revised edition of that treatise. But this issue is printed from entirely new manuscript and new plates. The illustrations, too, are largely new, all that have become antequated being discarded, thus making way for new pictures.

The author may be regarded, very justly so, too, as one of the most capable men in this special line of work, and this whether as a teacher or an author. The elder Keyes for many, many years occupied first place in line as a teacher and author. Like father, like son!

The author of this volume announces that it is intended, primarily, for student and general practitioner. But we think he has constructed a work that even the specialist cannot afford to ignore or to try and get along without. The topics dealt with relate to diseases of the urinary organs of both sexes, to diseases of the male genital organs, and to syphilis; and they are considered from both the medical and surgical viewpoints. Keyes is a graceful writer, but does not become prolix; he says what he aims to say, rounds his period, and stops; then takes up the next thought and deals with it in the same manner. The chapter on the social aspects of gonorrhea and its prevention, is a fine example of compact graceful rhetoric, and is, at the same time, a forceful presentation of the author's views. There are other chapters deserving similar comment; the book, indeed, is made up of such.

The ureter catheter, the cystoscope, and the *x*-ray are given their proper, their prominent places in diagnosis. They have revolutionised methods, caused advances in diagnosis, and transposed the arrangement of topics. This author, for example, throws overboard the anatomical arrangement of former years, and begins with principles of urology, then leads up to gonorrhea and prostatism; next, passes onward to inflammation, stone, tubercle, and to the various phenomena relating to hydronephrosis. A few chapters relating to neoplasms, injuries, anomalies, and some minor topics close the section on urology. Diseases of the genital organs which next follow are considered in nine chapters, and nowhere else have we found a clearer exposition of this topic. Especially so is the chapter on maladies involving the genital function.

The next topic is syphilis, twelve chapters, about one hundred pages being given over to its consideration. In no other place in the book and on no other subject is the author clearer or more self-accentuated than in the section on syphilis; in particular is he so when dealing with the treatment of syphilis. He accentuates the use of small doses of mercury rather than heroic ones, and makes it appear that it is equally efficient by the mouth, by inunction, or by injection, so long as the adequate amount is digested or absorbed.

The final section of the book deals with the operative surgery of genitourinary organs in the male which is considered in thirteen chapters. Useful hints or suggestions relating to almost all these operations are given, and many are illustrated in excellent fashion. We apprehend the adoption of this excellent treatise as a textbook in the majority of medical schools. It is not excelled for this purpose by any other treatise.

---

**Diagnostic Therapeutics.** By **Albert Abrams,** A.M., M.D. (Heidelberg), Consulting Physician to the Mount Zion Hospital and the French Hospital, San Francisco. Octavo, pp. 1039. With 198 illustrations. New York: Rebman Co. (Cloth, $5.00.)

The art of diagnosis has been pretty well exploited of late, both on the medical and surgical sides of the question. Butler, Wilson, Eisendrath, Webster, Wood, these and some others have discoursed upon methods of diagnosis within a recent period, and all have contributed materially to the advancement of medical science along these lines. It has remained for Abrams, however, to prepare a special work practically limited to pharmacotherapy.

The work, though, is not absolutely restricted to the employment of drugs in diagnosis, other methods having been added throughout the text for the purpose, the author states, of maintaining the continuity of the subject. Diagnosis through the employment of drugs we suppose disciples of Hahnemann would term "provings." Mistakes in diagnosis often lead to serious results, hence the importance of accuracy cannot be impressed too strongly on the young mind. Every rational method should be employed to ascertain the course of symptoms and to what lesions or disturbances they point.

Abrams's book is partly speculative and deals partly with established facts. Two chapters present miscellaneous topics, such as mistakes in diagnosis, factors in the etiology of disease, psychology of the patient, and so forth; and then in chapter three he presents the real purpose of the book—namely, to deal with diagnostic pharmacotherapy, giving a long list of drugs that enter into the question. In chapter four he takes up other methods than drug giving, such as abdominal supporters, massage, electrotherapeutic diagnostics, and a dozen or twenty other methods. In chapter five etiologic diagnostic therapeutics are considered. These embrace diatheses, puberty, menstruation and a few other topics. The diagnosis of visceral sufficiency forms the subject of chapter six, including the circulatory system, the respiratory apparatus, the glandular organs, the nervous system, muscular, pelvic, and vertebral insufficiency.

It is worthy of note that in this book of more than one thousand pages there are but six chapters in all, an unusually small number for such a large book. But the subheads and sideheads are so numerous that long chapters are not objectionable. Eight pages are given to bibliography in which three hundred and ninety references are recorded. The index is complete and the mechanical execution of the book is excellent.

---

**Preparatory and After-Treatment in Operative Cases.** By **Herman A. Haubold**, M.D., Clinical Professor in Surgery and Demonstrator of Operative Surgery in New York University and Bellevue Hospital Medical College, New York. Octavo, pp. 650. With 429 illustrations. New York and London: D. Appleton & Co. 1910. (Cloth, $6.00.)

The author of this excellent work has succeeded in placing before the practitioner in compact shape a mass of material that heretofore has not been published in separate form. These questions have been dealt with in surgical textbooks, but as a general rule in a more or less imperfect manner; or, at least, owing perhaps to crowded space, in an incomplete fashion. Here, however, we find a book quite as large as many of the earlier complete treatises on surgery, devoted only to the preparation of patients for operation and their care and management afterward. Of course, in the preparation of the patient as applied in the foregoing sentence is included also that of the operating room, the instruments, suture and ligature material, water and cleansing solutions, operator and assistants, nurses, together with the drainage, suturing, and dressing of operative wounds, though the latter more properly, perhaps, belong to post-operative attention.

The after-treatment includes shock and secondary hemorrhage; vomiting, thirst and pain; feeding after operations; the general care of wounds after operations; the adjustment of artificial limbs; skin grafting and miscellaneous matters pertaining to post-operative management.

Beginning with operations on the scalp, skull, and brain, the author takes up the preparation of the patient, and the after-care pertaining to almost every operation on the face, neck, thorax, spine, abdomen, stomach, intestines, liver, female pelvic organs, the vaginal route operations; operations on the rectum and anus; on the kidney and ureter; on the bladder and prostate; on the scrotum and penis; and, finally, operations on the extremities and miscellaneous operations. He shows how to prepare the patient for these operations and deals with the after-management in each condition. Such a work has become useful, if not necessary, by reason of the application of asepsis and antisepsis to operative surgery, as well as to injuries of the body. Haubold clings to the absurd word "celiotomy," which should be banished from literature. The book is especially useful to the general practitioner, yet every operator should possess it, and every student should study it.

